# Quantitative progression of retinitis pigmentosa by optical coherence tomography angiography

**DOI:** 10.1038/s41598-018-31488-1

**Published:** 2018-09-03

**Authors:** Ruben Jauregui, Karen Sophia Park, Jimmy K. Duong, Vinit B. Mahajan, Stephen H. Tsang

**Affiliations:** 1 Department of Ophthalmology, New York-Presbyterian Hospital, New York, NY USA; 2 Jonas Children’s Vision Care and Bernard & Shirlee Brown Glaucoma Laboratory, New York, NY USA; 3 Weill Cornell Medical College, New York, NY USA; 4 Department of Biostatistics, Columbia University, New York, NY USA; 5 Byers Eye Institute, Omics Laboratory, Department of Ophthalmology, Stanford University School of Medicine, Palo Alto, CA USA; 6 Department of Pathology & Cell Biology, Stem Cell Initiative (CSCI), Institute of Human Nutrition, College of Physicians and Surgeons, Columbia University, New York, NY USA

## Abstract

Optical coherence tomography angiography (OCT-A) is a non-invasive alternative to fluorescein angiography that allows for the study of the retinal and choroidal vasculatures. In this retrospective cohort study of 28 patients with retinitis pigmentosa (RP), we used OCT-A to quantify changes in perfusion density, foveal avascular zone (FAZ) area, and choriocapillaris blood flow over time and correlated these variables with ellipsoid zone (EZ) line width and best-corrected visual acuity (BCVA). Perfusion density decreased by 2.42 ± 0.62% per year at the superior capillary plexus (SCP) (P = 0.001) and 2.41 ± 0.76% per year at the deep capillary plexus (DCP) (P = 0.004). FAZ area increased by 0.078 ± 0.021 mm^2^ per year (P = 0.001) at the SCP and 0.152 ± 0.039 mm^2^ per year (P = 0.001) at the DCP. No changes were observed in the choriocapillaris blood flow. EZ line width had the strongest correlation to perfusion density at the SCP (r = 0.660 and 0.635, first and second visit, respectively, P = 0.001), while BCVA most strongly correlated with FAZ area at the SCP (r = 0.679 and 0.548, P = 0.001 and 0.003). Our results suggest that OCT-A is a useful tool for monitoring RP disease progression and may be used to measure retinal vascular parameters as outcomes in clinical trials.

## Introduction

Retinitis pigmentosa (RP) refers to a heterogeneous group of inherited rod-cone retinal dystrophies characterized by progressive visual field constriction and nyctalopia^1–3^. Its prevalence is estimated to be 1 in 4,000 people worldwide, while the vast majority of cases are inherited in an autosomal dominant, autosomal recessive, or X-linked manner^1,3^. With over 50 causative genes known to date, RP exhibits significant clinical and genetic heterogeneity, as a single mutation may cause a variety of clinical phenotypes and a variety of different mutations may cause the same syndrome^4^. The primary defect lies in the rod photoreceptors, which degenerate and lead to secondary cone cell death. Throughout the course of the disease, the retinal pigment epithelium and blood vessels are also affected, leading to additional clinical hallmarks of the disease such as attenuation of retinal vessels and intraretinal pigment migration.

With the advent of optical coherence tomography angiography (OCT-A), the study of the retinal and choroidal vasculatures has become more feasible. OCT-A serves as a non-invasive and ideal alternative to fluorescein angiography, as it not only is faster to obtain, but also avoids potential side effects of fluorescein angiography such as vomiting and hypersensitivity reactions^5^. OCT-A detects streaming blood flow and constructs an image of the retinal vasculature, allowing for the visualization of the superficial (SCP) and deep capillary plexus (DCP)^5^. The choriocapillaris is also visualized, but the small size and intersinusoidal spacing of its blood vessels cause the choriocapillaris to appear homogenous with bright areas representing blood flow^6^. The technology of OCT-A has been applied broadly to study vasculature changes in inherited retinal dystrophies, including RP, Stargardt disease, and choroideremia^7–11^.

It has previously been reported that perfusion density, defined as the total area of perfused vasculature per unit area in a region of measurement (also referred to as vessel density in some studies^12^), is decreased while the area of the foveal avascular zone (FAZ) is increased in patients with RP compared to controls^8^. This study aims to analyze and quantify changes in the retinal vasculature of patients with RP over time. In addition, we correlate these changes with the width of the ellipsoid zone (EZ) line, which is related to the size of a patient’s field of vision, and best-corrected visual acuity (BCVA). This work could not only have implications on the development of therapies for RP, but it could also establish the use of perfusion density and FAZ area as outcome measures for clinical trials and disease progression.

## Results

### Patients

In total, 28 patients (28 eyes) were analyzed for this study. Demographic characteristics of the patients are included in Table 1. The mean follow-up time was 1.3 ± 0.46 years. Complete descriptive statistics can be found in Supplementary Table S2.

**Table 1:** Demographic and genetic characteristics of the retinitis pigmentosa patients.

	No. (%)	Mean age (yr)	Mean follow-up time (yr)	No. (%) with CME
**Patients**	28	44.1 ± 18.45	1.3 ± 0.46	3/28 (10.7)
Males	17/28 (61)			
Females	11/28 (39)			
**Eye**				
OD	11/28 (39)			
OS	17/28 (61)			
**Inheritance**		**Genes with disease-causing variants (No**. **of patients)**		
ARRP	17/28 (61)	*PDE6A* (2), *USH2A* (2), *EYS* (2), *DHDDS* (1), *CERKL* (1), *KIZ* (1), *MERTK* (1), *TULP1* (1), Unknown (6)		
ADRP	8/28 (29)	*RP1* (3), *RHO* (2), *KLHL7* (2), *PRPF8* (1)		
USH	3/28 (11)	MYO7A (2), GPR98 (1)		

Data are summarized as mean ± standard deviation where appropriate. ARRP = autosomal recessive retinitis pigmentosa; ADRP = autosomal dominant retinitis pigmentosa; USH = Usher syndrome; CME = cystoid macular edema.

### Progression rates in the retinal and choroidal vasculatures

We observed a progression rate with quantitative OCT-A analysis in perfusion density at both the level of the SCP and DCP between the two visits. The perfusion density decreased over time at a rate of 2.42 ± 0.62% per year at the SCP (P = 0.001) and 2.41 ± 0.76% per year at the DCP (P = 0.004). We also observed a rate of progression in the FAZ area at both the SCP and DCP; at the SCP, the FAZ area increased at a rate of 0.078 ± 0.021 mm^2^ per year (P = 0.001), while it increased at a rate of 0.152 ± 0.039 mm^2^ per year at the DCP (P = 0.001). There was no significant progression rate observed for choriocapillaris blood flow (1.36 ± 1.23, P = 0.275). A rate of progression was also measured in the width of the EZ line, which decreased at a rate of 107.03 ± 13.67 µm per year (P < 0.001). BCVA decreased at a logMAR rate of 0.049 ± 0.021 per year (P = 0.026). These results are summarized in Table 2.

**Table 2:** Quantitative analyses of perfusion density, foveal avascular zone area, choriocapillaris blood flow, EZ line width, and BCVA at each visit and their yearly progression rate.

	Visit 1	P-value^a^	Visit 2	P-value^a^	Progression rate per year	P-value^b^	P-value^a^
Perfusion density, mean (%)							
SCP	33.4 ± 11.1	<0.001	30.4 ± 11.1	<0.001	−2.42 ± 0.62	0.001	0.986
DCP	24.5 ± 9.69		21.5 ± 9.57		−2.41 ± 0.76	0.004	
FAZ area, mean (mm^2^)							
SCP	0.345 ± 0.226	<0.001	0.430 ± 0.292	<0.001	0.078 ± 0.021	0.001	0.053
DCP	0.784 ± 0.389		0.944 ± 0.447		0.152 ± 0.039	0.001	
	**Visit 1**		**Visit 2**		**Progression rate per year**		**P-value** ^**b**^
Choriocapillaris blood flow							
Mean gray value	99.5 ± 11.1		98.8 ± 13.0		1.36 ± 1.23		0.275
EZ line width, mean (µm)	2674.9 ± 1766.8		2533.2 ± 1755.9		−107.03 ± 13.67		<0.001
BCVA, mean (logMAR)	0.28 ± 0.30		0.33 ± 0.33		0.049 ± 0.021		0.026

Data are summarized as mean ± standard deviation where appropriate. FAZ = foveal avascular zone; SCP = superior capillary plexus; DCP = deep capillary plexus; EZ = ellipsoid zone; BCVA = best-corrected visual acuity; logMAR = logarithm of the minimal angle of resolution. ^a^Calculated using a paired Student’s t-test to test for a difference between these values. ^b^Calculated using one-sample Student’s t-test to test for a difference from 0.

### Vascular differences between the superior and deep capillary plexus in the retina

The mean perfusion density at the SCP was compared against the mean perfusion density at the DCP for both visits, and their respective progression rates were also compared. Similar comparisons were performed for the FAZ area. At both visit 1 and 2, the mean perfusion density at the SCP (33.4 ± 11.1% and 30.4 ± 11.1% for visit 1 and 2, respectively) was observed to be different from the mean perfusion density at the DCP (24.5 ± 9.69 and 21.5 ± 9.57% for visit 1 and 2, respectively) (P < 0.001 for both visits). Furthermore, at both visits, the mean FAZ area at the SCP (0.345 ± 0.226 mm^2^ and 0.430 ± 0.292 mm^2^ for visit 1 and 2, respectively) was observed to be different from the mean FAZ area at the DCP (0.784 ± 0.389 mm^2^ and 0.944 ± 0.447 mm^2^ for visit 1 and 2, respectively) (P < 0.001 for both visits). No difference was seen between the progression rate of perfusion density at the SCP (−2.42 ± 0.62%) and that at the DCP (−2.41 ± 0.76%) (P = 0.986). Similar results were observed between the progression rate of FAZ area at the SCP (0.078 ± 0.021 mm^2^) and that at the DCP (0.152 ± 0.039 mm^2^) (P = 0.053). These results are summarized in Table 2.

### Correlations between vascular parameters in the retina and measures of visual function

EZ line width and BCVA were both correlated with perfusion density and FAZ area at the levels of the SCP and DCP as well as with choriocapillaris blood flow at both clinic visits (Table 3). EZ line width was observed to have a correlation with only perfusion density at the SCP and DCP, with the strongest correlation observed at the SCP (r = 0.660 and 0.635, P = 0.001 and 0.001 for the first and second visit, respectively). BCVA correlated with both perfusion density and FAZ area, but the strongest correlation was observed with FAZ area at the SCP (r = 0.679 and 0.548, P = 0.001 and 0.003 for the first and second visit, respectively). Choriocapillaris blood flow was not correlated with either EZ line width (r = −0.105 and −0.161, P = 0.594 and 0.413 for the first and second visit, respectively) or BCVA (r = −0.031 and 0.155, P = 0.876 and 0.432 for the first and second visit, respectively). These results are summarized in Table 3.

**Table 3:** Correlations of EZ line width and BCVA with perfusion density, foveal avascular zone areas, and choriocapillaris blood flow.

EZ line width (µm)	Perfusion density at SCP		FAZ area at SCP		Perfusion density at DCP		FAZ area at DCP		Choriocapillaris	
	Visit 1	Visit 2	Visit 1	Visit 2	Visit 1	Visit 2	Visit 1	Visit 2	Visit 1	Visit 2
r	0.660	0.635	−0.277	−0.274	0.537	0.552	0.073	0.157	−0.105	−0.161
P-value^a^	***0.001***	***0.001***	0.154	0.158	***0.003***	***0.002***	0.713	0.424	0.594	0.413
**BCVA (logMAR)**										
r	−0.434	−0.403	0.679	0.548	−0.472	−0.374	0.383	0.272	−0.031	0.155
P-value^a^	***0.021***	***0.034***	***0.001***	***0.003***	***0.011***	***0.050***	***0.044***	0.161	0.876	0.432

FAZ = foveal avascular zone; SCP = superior capillary plexus; DCP = deep capillary plexus; EZ = ellipsoid zone; BCVA = best-corrected visual acuity; logMAR = logarithm of the minimal angle of resolution; r = Pearson correlation coefficient. ^a^Calculated from the Pearson correlation coefficient. P-values indicating statistical significance are italicized and bold.

## Discussion

Changes in the retinal vasculature and hemodynamics have long been associated with RP. By fundoscopy, attenuation of the retinal vessels is seen long before bone spicule pigment formation. Histopathologic studies have revealed that the migration of retinal pigment epithelium (RPE) cells around inner retinal blood vessels stimulates deposition of extracellular matrix (ECM) that resembles ectopic Bruch’s membrane, and this perivascular ECM progressively thickens and occludes the lumen of the vessels, leading to compromised blood flow^13,14^. In accordance with the changes observed by histopathology, hemodynamics studies by magnetic resonance imaging (MRI) on RP mouse models have shown reduction in the choroidal and retinal blood flow^15,16^. Retinal blood flow velocities have also been found to be lower in patients with RP compared to the control group^17^. Recently, studies have confirmed by means of OCT-A that there are vascular alterations in patients with RP^8,18^. OCT-A has additionally shown that the density of the radial peripapillary capillary network, which correlates with the thickness of the retinal nerve fiber layer, is also reduced in RP patients as compared with normal controls^19^. The advent of OCT-A has ultimately allowed us to expand our knowledge regarding vascular changes in RP in a non-invasive and more efficient manner.

Unique to our study is the characterization of the progression of perfusion density and FAZ area at both the SCP and DCP in RP patients for an average of 1.3 years. As mentioned previously, Battaglia *et al*. had shown that, compared to controls, perfusion density is significantly lower at the SCP and DCP in RP patients^8^. In addition, the FAZ area at the DCP was significantly enlarged in RP patients^8^. In our study, we found that at any given visit, the SCP exhibited perfusion density and FAZ area values that significantly differed from those of the DCP (P < 0.001 for all comparisons). Another previous study analyzed normal subjects with OCT-A and quantified progression rates of perfusion density and FAZ area at the SCP and DCP^10^. They reported that perfusion density decreases at a rate of 0.26% per year at the SCP and 0.27% per year at the DCP. The rates of FAZ area increase were 0.0014 mm^2^ per year at the SCP and 0.0011 mm^2^ per year at the DCP. Given that these rates are much lower than the rates we report in our study, we suggest that RP causes a greater decrease in perfusion density and a faster increase in FAZ area than what normal subjects experience as they age. Furthermore, although we observe significant progression rates over time, the progression rate of perfusion density at the SCP was not different from that at the DCP (P = 0.986), with similar findings observed for the FAZ area (P = 0.053). In normal subjects, the progression rates of both FAZ area and perfusion density between the SCP and DCP were also similar, as described above^10^.

When analyzing blood flow at the choriocapillaris, no significant progression rate was observed between the two visits (P = 0.275). This result is in agreement with previous studies that have reported no difference in both the perfusion density of the choriocapillaris and the blood flow rate between RP patients and controls^8,11^. A study by Li *et al*. compared the retinal and choroidal blood flow in RP mouse models; although blood flow was decreased in both retinal and choroidal circulation compared to controls, choroidal blood flow changes were seen much later than retinal blood changes^15^. Results from our data and previous studies likewise suggest that changes in the choroidal vasculature occur later compared to those in the retinal vasculature^8,11^. Thus, longer follow-up may be needed to observe changes in the choriocapillaris and choroidal blood flow in RP patients. Given that the primary lesion in RP exists in either photoreceptors or the RPE, choroid hemodynamics may become affected during the late stages of disease.

Various non-invasive imaging techniques are used to monitor the progression of RP. One common technique involves measurement of the EZ line width. The EZ line correlates with the patient’s visual field boundaries. As such, multiple studies have used SD-OCT to visualize and measure EZ line width as a means of tracking disease progression^20–23^. Another imaging modality used to measure RP progression is short-wavelength fundus autofluorescence, which often reveals a ring of hyperautofluorescence in patients with RP^24^. This ring constricts over time, and the inner border has been found to correspond spatially with the EZ line^25,26^. In this study, we observed an annual decline of 107.03 ± 13.67 µm in EZ line width, a rate similar to what has previously been published^20^. In correlating the EZ line width to the variables we analyzed via OCT-A, we found that EZ line width correlated only to perfusion density at both the SCP and DCP. EZ line width did not correlate with FAZ area or choriocapillaris blood flow. Grunwald *et al*. had suggested previously that degeneration of the highly metabolic, oxygen-consuming photoreceptors leads to increased levels of oxygen in the inner retina, causing a vasoconstriction regulatory response in the retinal vasculature^27^. Further recent studies strengthen this hypothesis, as endothelin-1, a potent vasoconstrictor, has been shown to be significantly increased in the eyes of RP patients^28,29^. We believe that as disease progresses and photoreceptors degenerate, the EZ line, which is formed mainly by photoreceptor mitochondria, decreases in length, leading to increased vasoconstriction in the retinal vessels. This change appears as decreased perfusion density on OCT-A.

We also correlated BCVA with the variables analyzed by OCT-A. We found that BCVA correlates the strongest with the FAZ area at the SCP (r = 0.679 and 0.548, P = 0.001 and 0.003 for the first and second visit, respectively). This is similar to the results found in another study where OCT-A was used to estimate retinal blood flow in RP patients^11^. The authors found that BCVA correlated the strongest with the FAZ area in the SCP and with the parafoveal flow density in the DCP. In our study, we measured perfusion density in place of blood flow at the SCP and DCP. Of note, BCVA also correlated with perfusion density at both the SCP and DCP in our study. Nevertheless, the strongest correlation was observed with the FAZ area at the SCP. Other studies have also compared morphological vascular changes in RP and correlated them to functional parameters. In a study with RP patients by Toto *et al*., for example, macular capillary density was imaged with OCT-A and correlated with macular function as measured by multifocal electroretinogram (mfERG)^18^. The authors found that the density of vessels in the choroid and retina was decreased, which correlated with a decline in ganglion cell complex layer thickness and macular function based on mfERG^18^.

Although there is no current treatment for RP, different treatment modalities such as gene therapy, neurotrophic growth factors, and retinal prostheses are being studied and have shown promising and encouraging results^30–34^. Regardless of the treatment modality, however, a healthy vascular supply is needed to maintain and support the various retinal cells, including photoreceptors and cells of the inner retina. Any extent of degeneration of the retinal and choroidal vasculatures may limit the impact of these promising therapies. For this reason, it is important to study how these vasculatures change throughout the course of RP.

Limitations to this study include the relatively short length of follow-up and the inherent disadvantages of using OCT-A. The average length of time between visits in this study was 1.3 years; given the recent Food and Drug Administration (FDA) approval of OCT-A in 2015, it is challenging to gather patients with longer follow-up that qualify for the study. Furthermore, given that our study only includes two time points per patient, our data appears to indicate that the OCT-A parameters decline in a linear fashion. Future studies with longer follow-up and a greater number of time points should address whether these OCT-A parameters indeed decline linearly throughout time, as the inclusion of more time points may instead reveal that they in fact fit an exponential regression model. Finally, in order to produce an accurate analysis, only patients with high-quality OCT-A scans were analyzed. Due to the fact that the acquisition of high-quality OCT-A images is heavily dependent upon the patient’s ability to fixate, most patients with advanced RP were excluded from the study as they lack fixation due to poor vision. This limits the possibility of studying changes in the retinal and choroidal vasculature in patients with advanced RP, a stage where one might observe significant changes.

To our knowledge, our study is the first to follow RP progression through OCT-A over time. We observed that at the SCP, perfusion density decreases at a rate of 2.42 ± 0.62% per year, while the FAZ area increases at a rate of 0.078 ± 0.021 mm^2^ per year. At the DCP, perfusion density decreases at a rate of 2.41 ± 0.76% per year, while the FAZ area increases at a rate of 0.152 ± 0.039 mm^2^ per year. In addition, we found that the EZ line width had a significant correlation with perfusion density at both the SCP and DCP, although it correlated the strongest at the SCP. Moreover, we found that BCVA correlated the strongest with the FAZ area at the SCP. This study is significant because we observe progressive vascular changes in the retina, which could have implications on emerging therapies for RP. Furthermore, perfusion density and FAZ area could be implemented as important variables and measures of progression in future clinical trials.

## Methods

### Patients and Clinical Examination

All study procedures were defined and informed patient consent for study participation was obtained as outlined by the protocol #AAAR0284 approved by the Institutional Review Board at Columbia University Medical Center. The study adhered to the tenets of the Declaration of Helsinki. None of the data presented in this study, including images and genetic testing results, is identifiable to individual patients. A retrospective review of 100 patients with a clinical diagnosis of retinitis pigmentosa by an inherited retinal disease specialist (SHT) was conducted at the Department of Ophthalmology at Columbia University. The clinical diagnosis was made based on presenting symptoms, family history, fundus examination, and full-field electroretinography (ffERG) and subsequently supported by clinical imaging and/or genetic testing. The inclusion criteria for this study were the diagnosis of RP along with clear media and adequate fixation to allow for high-quality imaging. In addition, each patient was screened for a history of two visits in our office at least 6 months apart consisting of a complete ophthalmic examination by a retinal physician (SHT). Ophthalmic examinations included a slit-lamp and dilated funduscopic examination, best corrected visual acuity (BCVA), fundus autofluorescence (FAF, 488 nm excitation), spectral domain optical coherence tomography (SD-OCT), and OCT angiography (OCT-A). The exclusion criteria precluded patients affected by any other ocular disorder or an advanced form of RP. Furthermore, eyes with poor OCT-A images that exhibited a signal strength index lower than 7 out of 10 were excluded from analysis. One eye from each patient was chosen for analysis based on image quality and inclusion/exclusion criteria.

### Clinical Characterization

Imaging across all modalities was conducted after pupil dilation (>7 mm) with phenylephrine hydrochloride (2.5%) and tropicamide (1%). Horizontal foveal SD-OCT scans measuring 9 mm and fundus autofluorescence (FAF, 488 nm excitation) were acquired with the Spectralis HRA + OCT (Heidelberg Engineering, Heidelberg, Germany). OCT-A 3 × 3 mm scans centered on the fovea were obtained using the Zeiss AngioPlex Cirrus HD-OCT 5000 (Zeiss Meditec. Inc, Dublin, California, USA). This instrument has an A-scan rate of 68 KHz scans and wavelength-scanning light of 840 nm. Each OCT-A contains 245 B-scans (each B-scan contains 245 A-scans). To image the motion of moving erythrocytes, 4 OCT scans are performed at the same location, assisted by eye tracking technology. Automated segmentation of full-thickness retina scans into superficial and deep inner retinal vascular (capillary) plexus (SCP and DCP, respectively) and choriocapillaris by the machine was performed. The SCP is bounded by the internal limiting membrane and the inner plexiform layer, containing vasculatures from the nerve fiber, ganglion cell, and inner plexiform layers^35,36^. The DCP is bounded by the inner plexiform and outer plexiform layers, containing vasculatures from the inner nuclear and outer plexiform layers^35,36^.

### Image Analysis

**Figure 1:**
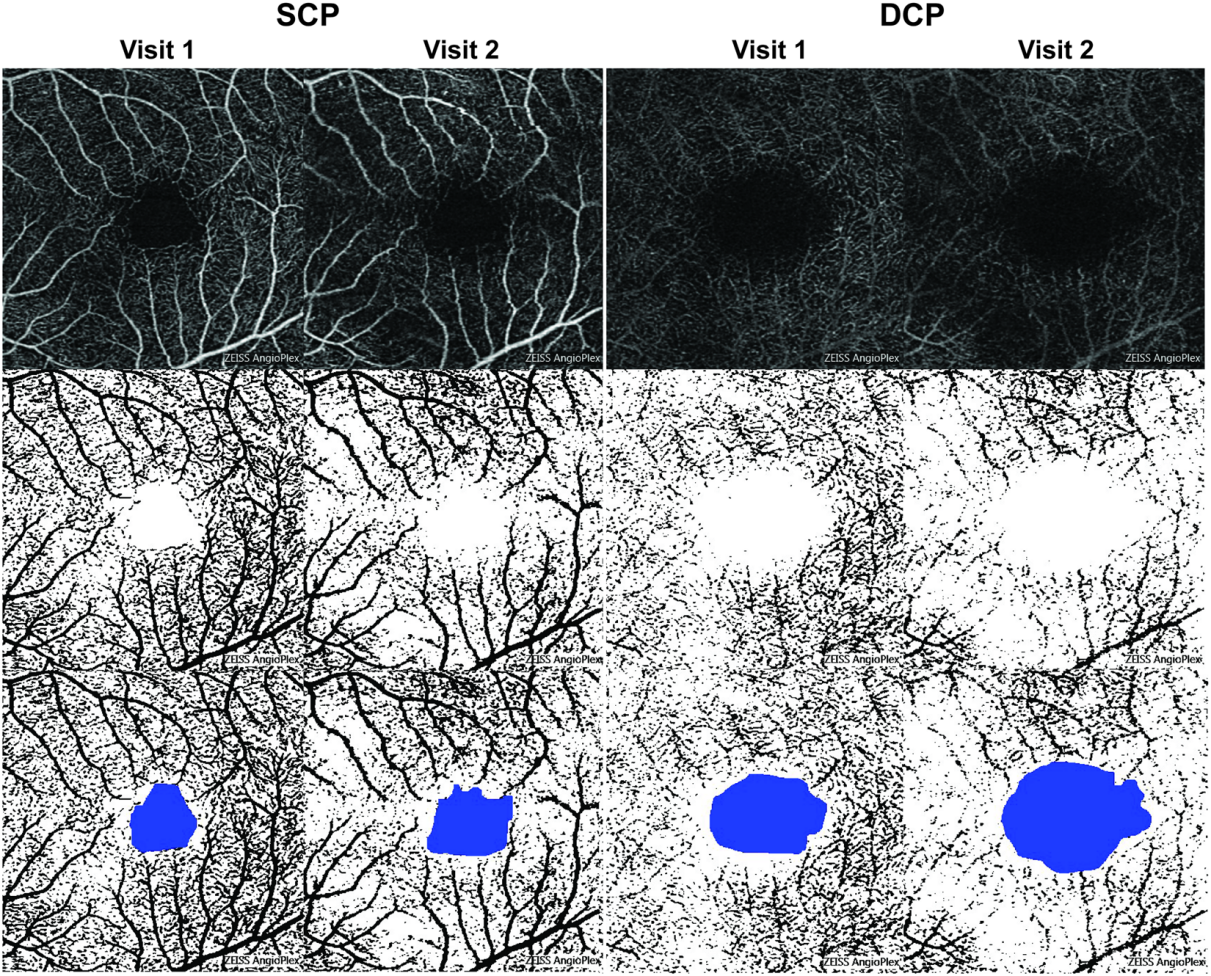
Changes in the retinal vasculature of a patient with retinitis pigmentosa on optical coherence tomography angiography. Optical coherence tomography angiography (OCT-A) images and demarcation of the foveal avascular zone (FAZ) area of the superior capillary plexus (SCP) and deep capillary plexus (DCP) from visit 1 and 2 are shown from a patient with retinitis pigmentosa. Raw OCT-A images (first row) were converted into binarized and skeletonized images (second row) depicting blood vessels in black and background in white. The binarized and skeletonized images were used to quantify FAZ area, outlined and shaded in blue, as well as perfusion density (third row). The binarized and skeletonized OCT-A scans in the third row are duplicates of those in the second row. Compared to visit 1, perfusion density is lower and FAZ area is higher in visit 2.

The analysis of the OCT-A images was performed as previously described^7,8^. All of the 3 × 3 mm OCT-A images were collected and exported from the system as a Joint Photographic Experts Group (JPEG) file into the National Institutes of Health ImageJ 1.8.0 (National Institutes of Health, Bethesda, Maryland, USA) image-processing software. The image was converted from 8-bit into red green blue (RGB) color type and was subsequently split into the three channels. We chose the red channel as reference as previously performed^7,8^. The adjust threshold tool was set to default and applied, with the dark-background option selected. This tool sets lower and upper threshold values (60–255 in this study) and segments the grayscale images into features of interest and background. After processing, the images were converted to RGB. The foveal avascular zone (FAZ) area was manually outlined using the free-hand selection tool, and its dimensions were expressed in millimeter squared using a method previously described^9^. The FAZ area was colored in blue. Black pixels were considered vessels, white pixels were background, and blue pixels were the FAZ area. Perfusion density, defined as the total area of perfused vasculature per unit area in a region of measurement, was calculated as the ratio of black pixels to the total number of pixels, with the blue FAZ area pixels excluded. This method was used to calculate perfusion density in both the SCP and DCP (Fig. 1). To analyze the choriocapillaris, we used the mean gray value (MGV) of the unprocessed grayscale 8-bit OCT-A images of the choriocapillaris as an objective proxy for blood flow as previously described^6^. A high degree of blood flow through the choriocapillaris is depicted by high MGV and image brightness. SD-OCT scans were acquired as a single horizontal slice through the fovea per patient, and measurement of the ellipsoid zone (EZ) line width from these scans was performed. Both perfusion density image analysis and EZ line width measurements were performed by two independent graders (RJ and KSP).

### Statistical analyses

The statistical analyses were performed using the Stata 12.1 (StataCorp, College Station, Texas, USA) software. The Pearson correlation was calculated for the measurements of both independent graders (see Supplementary Table S1). Given the high correlation between the two graders, the average of the two values obtained from each of the graders was calculated and used for subsequent analysis. Statistical analysis included descriptive statistics for demographics, logMAR BCVA, EZ line width, and OCT-A measurements (perfusion density for the SCP and DCP, FAZ area for the SCP and DCP, and choriocapillaris blood flow) for both visits. Change over time, defined as the difference in values obtained between the follow-up and baseline visits divided by the length of follow-up, was calculated for these variables. One-sample Student’s t-test was used to determine whether the mean change-over-time variables were different from 0. A paired Student’s t-test was used to compare the mean values at each visit and the perfusion density and FAZ area progression rates between the SCP and DCP. Individual bivariate analyses were performed to correlate each OCT-A measurement value with the EZ line width and logMAR BCVA. In all of the statistical analyses, we defined statistical significance as a P-value of less than 0.05.

## Electronic supplementary material

Tables S1 and S2

## Data Availability

The datasets generated during and/or analyzed during the current study are available from the corresponding author on reasonable request.
