# Supplementary material for: Quantitative progression of retinitis pigmentosa by optical coherence tomography angiography
Source: Sci Rep. 2018 Sep 3;8:13130. doi: 10.1038/s41598-018-31488-1 (PMC6120860; doi:10.1038/s41598-018-31488-1)
Supplement: Supplementary file 1 — Tables S1 and S2 [file 41598_2018_31488_MOESM1_ESM.docx]

**Quantitative progression of retinitis pigmentosa by optical coherence tomography angiography**

Ruben Jauregui^1,2,3^, Karen Sophia Park^1,2^, Jimmy K. Duong^4^, Vinit B. Mahajan^5^, Stephen H. Tsang^1,2,6 §^

^1^ Department of Ophthalmology, New York-Presbyterian Hospital, New York, NY, USA

^2^ Jonas Children’s Vision Care and Bernard & Shirlee Brown Glaucoma Laboratory, New York, NY, USA

^3^ Weill Cornell Medical College, New York, NY, USA

^4^ Department of Biostatistics, Columbia University, New York, NY, USA

^5^ Byers Eye Institute, Omics Laboratory, Department of Ophthalmology, Stanford University School of Medicine, Palo Alto, CA, USA

^6^ Department of Pathology & Cell Biology, Stem Cell Initiative (CSCI), Institute of Human Nutrition, College of Physicians and Surgeons, Columbia University, New York, NY, USA.

^§^ Corresponding author

Running title: OCT-A progression in retinitis pigmentosa

Address Correspondence: Stephen H. Tsang, MD, PhD, Harkness Eye Institute, Columbia University Medical Center, 635 West 165th Street, Box 212, New York, NY 10032, Phone: (212) 342-1189 / Fax: 212-305-4987 / Email: [sht2@cumc.columbia.edu](mailto:sht2@cumc.columbia.edu)

**Supplementary Information**

**Table S1.** Correlation of measurements for the two independent graders

|  | **PD at SCP** | | **FAZ at SCP** | | **PD at DCP** | | **FAZ at DCP** | | **EZ line width** | |
| --- | --- | --- | --- | --- | --- | --- | --- | --- | --- | --- |
| **Pearson Correlation** | **Visit 1** | **Visit 2** | **Visit 1** | **Visit 2** | **Visit 1** | **Visit 2** | **Visit 1** | **Visit 2** | **Visit 1** | **Visit 2** |
| r | 0.996 | 0.993 | 0.960 | 0.952 | 0.988 | 0.999 | 0.932 | 0.920 | 0.996 | 0.997 |
| P-value | <0.001 | <0.001 | <0.001 | <0.001 | <0.001 | <0.001 | <0.001 | <0.001 | <0.001 | <0.001 |

PD = perfusion density; FAZ = foveal avascular zone; SCP = superior capillary plexus (SCP); DCP = deep capillary plexus; EZ = ellipsoid zone; r = Pearson correlation coefficient.

| **N** | **Mean (yr)** | **Standard Deviation** | **Quartile** | | | | |
| --- | --- | --- | --- | --- | --- | --- | --- |
|  |  |  | **Minimum** | **25^th^** | **Median** | **75^th^** | **Maximum** |
| 28 | 1.3 | 0.46 | 0.68 | 0.92 | 1.09 | 1.82 | 2.04 |

**Table S2**. Distribution of follow-up time between the two visits
